# Protocol-Guided Phase-1 Cardiac Rehabilitation in Patients with ST-Elevation Myocardial Infarction in A Rural Hospital

**DOI:** 10.4103/1995-705X.73209

**Published:** 2010

**Authors:** Abraham Samuel Babu, Manjula Sukumari Noone, Mohammed Haneef, Shijoy M. Naryanan

**Affiliations:** 1 Department of Rehabilitation, CSI Mission Hosptial, Codacal, Kerala, India; 2 Department of Physiotherapy, Manipal College of Allied Health Sciences, Manipal University, Karnataka, India; 3 Department of Internal Medicine, CSI Mission Hospital, Codacal, Kerala, India; 4 Department of Medicine, Koyili Hospital, Kannur, Kerala, India

**Keywords:** Acute coronary syndrome, cardiac rehabilitation, coronary artery disease, physical therapy

## Abstract

**Aims:**

Phase-1 Cardiac Rehabilitation (CR) is an important part in the treatment of patients with ST-Elevation Myocardial Infarction (STEMI). Lack of literature in the rural Indian setting led to the design of this study.

**Setting and Design:**

Secondary care rural hospital, non-randomized experimental study.

**Materials and Methods:**

Fifteen historical controls and 15 prospectively enrolled patients between January 2007 and December 2007. The prospectively enrolled patients received the phase-1, exercise-based, protocol-guided CR. At discharge, the six-minute walk test (6MWT) distance, Borg’s Rating of Perceived Exertion (RPE) after the 6MWT, time to return to baseline parameters after the 6MWT, and complications were assessed.

**Statistical Analysis used:**

Independent t-test and the Mann Whitney test.

**Results:**

Statistically significant (*P* < 0.01) differences in ratings of perceived exertion (RPE) and time to return to baseline parameters post the 6MWT were seen in the experimental group ((2 vs. 4 and 5.47 vs. 7.93 minutes, respectively). No significant changes in the 6MWT distance between the groups were noticed (470±151.76 m and 379±170.70 m, respectively). No adverse events during the 6MWT and the phase-1 CR were observed.

**Conclusion:**

Protocol-guided, phase-1 CR produces a much faster return of heart rate and blood pressure to baseline following the 6MWT, without producing a great rise in the RPE during the 6MWT, which suggests a training benefit among these patients. The 6MWT can be safely administered in this rural population. However, larger studies will be required to validate these results.

## INTRODUCTION

Cardiac Rehabilitation (CR) is an interdisciplinary team approach to patients with functional limitations secondary to heart disease.[1] The World Health Organization (WHO) has defined CR as the sum of activities required to favorably influence the underlying cause of the disease, as well as the best possible physical, mental, and social conditions, so that they may, by their own efforts, preserve or resume, as normal a place as possible in the society. Rehabilitation cannot be regarded as an isolated form of therapy, but must be integrated within the entire treatment.[2]

Cardiac Rehabilitation has come a long way from the initial ‘bed-rest’ approach used to treat ST-elevation myocardial infarction (STEMI). Advances in medical technology have led to a shorter duration of hospitalization. Pioneering work done by Wenger *et al*.[3] developed the formulation of phase-1 CR, which along with recent medical advances, has led to a shorter duration of hospitalization.

Exercises have been seen to be beneficial for patients with *coronary artery disease* (CAD), however, vigorous exercises can have disastrous effects.[4] Therefore, it is essential to have a gradation of exercises for a patient with CAD, in order to prevent sudden cardiac complications during the exercise.

Cardiac Rehabilitation has been conventionally divided into four phases. Phase-1 involves the hospitalized period of the patient following an acute MI, phase-2 is the immediate post discharge period, phase-3 is the stage of a structured exercise program, and phase-4 is the maintenance phase.[5] CR has a set of core components that should be included into every program. These components include baseline patient assessment, nutritional counseling, risk factor modification, psychosocial interventions, physical activity counseling, and exercise training.[6] Early mobilization has helped to decrease the effects of bed rest, enabling the patients to return to their activities of daily living (ADL), within the limits of the disease, to identify patients at risk of cardiovascular and physical impairments, and finally to prepare the patient and the support system at home to optimize recovery.[7]

The American College of Sports Medicine (ACSM) recommendations for the intensity in phase-1 CR among post MI patients include, training the patient up to a heart rate of 120 beats / minute, guided by symptoms of chest pain and dyspnea (Borg’s rating of perceived exertion < 13)[7] and interval training with bouts of exercises lasting from three to five minutes or as tolerated, interspersed with adequate rest periods in order to achieve an exercise / rest ratio of 2 : 1. Following an acute coronary event, phase-1 CR is important for helping the patient to recover. It consists of medical evaluation, reassurance, and education regarding CAD, correction of cardiac misconceptions, risk factor assessment, mobilization, and discharge planning. Mobilization of the patient may include graded exercises, walking, and stair climbing.[8] However, there is no protocol regarding the progression of physical activity and exercises during this phase, as each treatment is individualized. In India, there is a lack of published work on CR both at the tertiary and rural level. This study intends to demonstrate the beneficial effects of a protocol-guided, phase-1 rehabilitation for patients with non-complicated STEMI in a rural center.

## MATERIALS AND METHODS

A non-randomized experimental study was carried out on 30 patients with acute coronary syndrome (ACS) with ST elevation or STEMI receiving phase-1 CR. They were divided into two groups; group 1 — experimental group and group 2 — historical control. Patients having ACS without ST elevation, unstable angina, new onset *left bundle branch block* (LBBB), and heart failure were excluded. All STEMI were managed as per the guidelines recommended by the American Heart Association (AHA) and American College of Cardiology (ACC).[9] A total of 15 patients fulfilled these criteria from June 2007 to December 2007. These patients were compared against 15 historical controls who did not receive phase-1 CR, but were administered the six minute walk test (6MWT) prior to discharge. This design was chosen in order to avoid ethical issues of withholding CR from deserving patients.

The protocol followed for phase-1 rehabilitation of patients with STEMI was developed in the hospital after modifications and trials were conducted for a year. Phase-1 CR was administered to the patients under supervision, keeping in mind the Rate Pressure Product (RPP), heart rate, blood pressure, Modified Borg’s Rating of Perceived Exertion (RPE), and cardiac symptoms. The protocol developed for a five-day hospitalization period, following STEMI, is given in Table 1. The principles followed were low intensity, short duration, early mobilization, and progression of intensity as tolerated. These were in accordance with the recommendations by the ACSM.[7] Group-1 received phase-1 rehabilitation as guided by the protocol. This was implemented by the physical therapist. Specific instructions and precautions, if any, were intimated by the treating physician. The control group received CR in the form of ambulation, which was physician-guided. Ethical clearance and informed consent was obtained prior to initiation of rehabilitation.

**Table 1:** Phase-1 protocol

Level 1: (Complete Bed Rest — Day of admission)
Relaxation
Breathing exercises
Active range of motion exercises (Ankle foot movements and finger and wrist movements) performed five times, thrice daily.
Level 2: (Partial Bed Rest — Days 1 and 2)
Sitting (1 – 2 hours / day) and self-feeding Relaxation Breathing exercises Active range of motion exercises to hip and knee (five repetitions, thrice day) Sitting — arm bending / stretching up / bending (five repetitions, thrice a day) Level 2 (a) — progress sitting time (3 – 4 hours / day) Independent in toileting (bedside) Alternate heel drags Static quadriceps and glutei (do not hold breath) Static + spinal extension (five repetitions, thrice a day)
Level 3: (Up and About — Days 3 – 5)
STOP relaxation Level 2(b)— progress exercises to 10 repetitions *Walk within room (thrice a day) Standing — Upper limb flexion (five repetitions thrice a day) Level 3(a) Walk-standing — lower limb flexion (five repetitions thrice a day) Stride-standing — hip and knee flexion (five repetitions thrice a day) *Walking outside the room (thrice a day) Level (3b) Bend standing — elbow circling Trunk bending *Walking outside the room with arm swings Climbing one flight of steps

Note: This is only a guide and is not to be followed strictly, but rather modified to suit each patient.

* Walking distance is determined by the patient’s ability to walk without any angina, dyspnea, tachycardia, fall in BP, and S3 gallop.

All patients were made to perform a sub-maximal stress test using the 6MWT, prior to discharge, according to the American Thoracic Society guidelines.[10] The 6MWT was carried out by the physical therapist in charge of rehabilitation. Beta blockers were not stopped prior to testing because of the early timing of the test following the cardiac event. Therefore, the RPE was monitored regularly during the test. The end points of the RPE were taken to be between 7 and 8.[11] Blood pressure (BP) and Heart Rate (HR) were measured before and after the test.

Following the 6MWT, an unsupervised phase-2 exercise program, dietary advice, patient education, identification of symptoms (red flags), risk factor modification, proper technique of taking sublingual Glyceryl Tri-Nitrate (GTN), and the importance of compliance with medical treatment were explained to the patient and their relatives by the physical therapists. Medical follow-up and the appropriate change in medications were done by the consulting physicians.

### Outcome measures

- 6WMT distance at discharge
- Modified Borg’s rating of perceived exertion (RPE)
- Exercise-induced ischemia
- Time to return of baseline (TTB) vitals after 6MWT
- Percentage of age-predicted target heart rate (%THR) achieved during the 6MWT
  (THR is normally achieved only from formal exercise testing and as such facilities are not available in this center, a percentage of the age-predicted target heart rate is used).
- Complications with the protocol

### Statistical analysis

All data was entered and analyzed with SPSS 16.0. Nominal data has been summarized using mean and standard deviation. Independent t-tests were done for tests of significance, for 6MWT and TTB. Non-normal data, that is, %THR and RPE, were analyzed using the Mann-Whitney test. The sample size for this pilot study was estimated to be 30. A formal estimation of the sample size could be made from this data for further studies.

## RESULTS

Of the 30 patients, five were women and the rest men. In the group that received CR, two were women and 13 were men [Table 2]. The average age of the persons who received CR was 47 ± 10 years and for the control group it was 48 ± 11 years. Out of the 15 patients in the experimental group with STEMI, two were treated with streptokinase (SK), while the rest were managed with heparin. In the control group, however, eight were treated with SK. No significant differences were seen between the groups at admission (p > 0.05).

**Table 2:** Demographic details

	Cardiac rehabilitation (n = 15)	Control (n = 15)	Total (n = 30)
Age (mean ± SD)	47 ± 10.9	52.4 ± 13.2	49.7 ± 12.2
Sex (Male / Female)	13 / 2	12 / 3	25 / 5
AWMI (n, %)	8 (26.7)	8 (26.7)	16 (53.3)
IWMI (n, %)	5 (15.6)	6 (20)	11 (36.7)
LWMI (n, %)	2 (6.7)	0	2 (6.7)
ASMI (n, %)	2 (6.)	1 (3.3)	3 (10)
RV (n, %)	0	1 (3.3)	1 (3.3)
Lysis (n, %)	6 (40)	8 (46.7)	14 (45.2)
Successful lysis (n)	5	7	12
Heparin (n, %)	9 (60)	7 (46.7)	16 (51.6)

An increase in the average distance walked in those receiving phase-1 CR can be seen Table 3. The distance walked in the CR group was 470 ± 151 meters (m), while in the control group it was 379 ± 170 m. This difference was, however, not statistically significant (p = 0.13). The RPE during the 6MWT in the group receiving the protocol-guided CR was 2, while in the control group it was 4. This was statistically significant when compared between the groups (p < 0.001). Age predicted %THR achieved was higher in the CR group than in the control group (p = 0.012). The average time taken for the vital signs to return to normal post 6MWT was lower in the group receiving CR (5.4 minutes) when compared to the control group (7.9 minutes). This was found to be significant (p = 0.001). No complications or mortality from the use of the protocol was observed during hospitalization. There was no change in the length of stay observed between the groups (p > 0.05).

**Table 3:** Results of the six minute walk test

	Cardiac rehabilitation (n = 15)	Control (n = 15)	*P* value
Distance (mean, SD)	470 ± 151.76	379 ± 170.79	0.13
RPE (median, range)	2 (4.2)	4 (5.4)	< 0.001†
Time to baseline in minutes (mean, SD)	5.47 ± 1.73	7.93 ± 2.01	0.001
Age-predicted %THR (median, range)	68.23 ± 5.28	62.66 ± 6.51	0.012†
Exercise-induced ischemia, n (%)	14 (82.35%)	11 (73.33%)	

† Mann-Whitney test

## DISCUSSION

A total of 15 patients with STEMI received phase-1 CR with the designed protocol. Historical controls (15) for this group were taken retrospectively and then compared with those receiving the protocol.

Cardiac rehabilitation has long been recognized as integral to the comprehensive management of patients, after hospitalization for MI.[12] CR has been shown to decrease the mortality by 20 – 24% and also to be highly cost effective.[12,13]

Submaximal stress testing is useful in the clinical setup for pre-discharge, post MI evaluations, and also for appropriate recommendations regarding physical activity.[11] The 6MWT has been used as a good indicator for assessing the functional capacity of persons, for pre-treatment and post-treatment comparisons, and as a predictor of mortality and morbidity.[14] Submaximal exercise testing appears to have a greater applicability to physical therapists in their role as clinical exercise specialists.[15] The 6MWT is a simple, inexpensive, and easy-to-complete test. The 6MWT distance corresponds to functional activities used in daily activities.[16] The use of a standard time rather than a predetermined distance provides a better test of endurance. The tests allow the individual to set her or his own pace and stop if necessary.[15] This series shows that the 6MWT can be conducted safely on post STEMI patients without any adverse complications. The non-significant change in the 6MWT distance could be attributed to the co-existing, age-related, musculoskeletal changes in the appendicular and axial skeleton, which would hamper the distance walked and also the small sample studied. However, the 6MWT distance can be used as a good indicator to regulate the walking time during a home-based, phase-2 CR program.

The RPE increase was significantly lower in the group where phase-1 CR was provided. This shows that there are short-term benefits of exercises during phase-1. This along with the decreased time for the vitals to return to baseline post 6MWT, all point to the importance of exercises during phase-1, for helping the body’s response to physical activity immediately after a coronary event. Even though there was no change in the length of stay in the hospital, physical and functional benefits were seen among these patients. Such a protocol-guided program would also facilitate better activity levels during phase-2, with no complications. This hypothesis would, however, need to be tested on a larger, well-controlled trial.

The age-predicted % THR achieved during the 6MWT was 69 and 63% in the experimental and control groups, respectively. This was found to be statistically significant (p = 0.012). However, this measurement of THR could not be considered accurate, as the patients were still on beta-blockers and would therefore need to be interpreted with caution. The RPE is a better tool to be considered, while assessing the progression of exercises.

The time taken for the vitals to return to baseline following the 6MWT is lower in the CR group (5.46 minutes) than in the control group (7.93 minutes). This difference is statistically significant (p = 0.002). This finding suggests that even a progressive exercise program of five days is sufficient to bring about the minimal effects of training in patients with STEMI. However, the duration of carry-over is not known. These findings, however, will require further research on a larger sample size, with follow-up. Thus, it can be stated that early mobilization with this new protocol at the community level, supports the previous studies published.[17] This, however, contradicts the study done by Oldridge *et al*.[18] and Sivarajan *et al*.[19] where they found no additional physiological or behavioral benefits with structured in-hospital exercise programs following an MI. All the same, with the current advances in medicine, the benefits and costs of a phase-1 CR program needs to be analyzed.

An almost equal occurrence of exercise-induced ischemia was observed in the experimental group (12 / 15) and the control group (11 / 15). The reason for this finding could be that vigorous physical exertion increases myocardial oxygen demand and simultaneously shortens diastole and coronary perfusion time, which may induce myocardial ischemia.[20] This further stresses the importance of a gradual progressive exercise program during phase-1 CR. Out of the 12 persons in the experimental group, 10 completed the 6MWT, while in the control group only four of the 11 were able to complete the test. Therefore, even though the occurrence of exercise-induced ischemia is almost the same in both the groups; the duration required for the onset of these changes is lesser in the experimental group. This further supports the use of a protocol-guided CR program.

Brown *et al*.,[21] in a meta-analysis found that there was a decrease in all-cause and total mortality when compared to the exercise-only intervention and usual care (RR 0.76 and 0.73, respectively). Even though mortality was not followed up as a primary outcome, no in-hospital mortality was observed as a result of this rehabilitation program.

Limited technology has forced the authors to rely on vital signs (pulse and BP), auscultation, and RPE as indicators for stressful situations during exercise. The authors acknowledge the drawbacks in this study, but find it important to highlight that this may have significant relevance at the community level in developing countries, where advanced interventional cardiology centers do not exist. Therefore, such a program would facilitate the rehabilitation of persons admitted with ACS. However, further investigations of this protocol with appropriate physiological measurements will be essential. The cost effectiveness of this program has not been analyzed. Prospective, blinded, randomized control trials on a large sample size will be required to draw definite conclusions regarding the use of this protocol-assisted, phase-1 CR program.

## CONCLUSION

Beneficial effects of exercises in this phase were seen in terms of return to baseline of HR following the 6MWT, onset of exercise-induced ischemia, and RPE during the 6MWT. Monitoring of pulse, BP, and RPE are sufficient for the assessment of the patient’s condition during the exercises. The 6MWT can be used safely as a submaximal stress test in the community following an STEMI, as a pre-discharge evaluation tool, to prescribe appropriate physical activity.
